# Supplementary material for: Predicting vaccine effectiveness against severe COVID-19 over time and against variants: a meta-analysis
Source: Nat Commun. 2023 Mar 24;14:1633. doi: 10.1038/s41467-023-37176-7 (PMC10036966; doi:10.1038/s41467-023-37176-7)
Supplement: Supplementary file 1 — Supplementary Information [file 41467_2023_37176_MOESM1_ESM.pdf]

Predicting vaccine effectiveness against severe COVID-19 over time and against variants: a meta-analysis

Deborah Cromer[1*], Megan Steain[2,3], Arnold Reynaldi[1], Timothy E. Schlub[1,4], Shanchita Khan[1], Sarah C. Sasson[1], Stephen J. Kent[5, 6], David S. Khoury[1], Miles P. Davenport[1]

1. Kirby Institute, University of New South Wales, Sydney, Australia
2. Sydney Institute of Infectious Diseases and Charles Perkins Centre, Faculty of Medicine and Health, The University of Sydney, Sydney, Australia.
3. School of Medical Sciences, Faculty of Medicine and Health, The University of Sydney, Sydney, Australia.
4. Sydney School of Public Health, Faculty of Medicine and Health, University of Sydney, Sydney, Australia
5. Department of Microbiology and Immunology, University of Melbourne at the Peter Doherty Institute for Infection and Immunity, Melbourne, Australia
6. Melbourne Sexual Health Centre and Department of Infectious Diseases, Alfred Hospital and Central Clinical School, Monash University, Melbourne, Australia.

Supplementary Methods

Literature search for data on vaccine effectiveness

In order to identify studies to be used in this meta-analysis, we searched PubMed for papers indexed between inception and 2nd March 2022 (Pubmed search: (SARS-CoV-2 OR COVID-19) AND (followup OR waning OR duration OR durable) AND (protection OR efficacy OR effectiveness)) and also monitored other public sources of information such as Twitter and medRxiv.

To be included in our analysis, studies must have included data on the efficacy or effectiveness against a defined symptomatic COVID-19 and/or severe COVID-19 clinical endpoint, of a primary COVID-19 vaccine schedule in humans, compared to an unvaccinated

control population and over time (i.e. present a time series of efficacy/effectiveness). In addition, these studies must have efficacy/effectiveness data reported:

(i) for a single vaccine (or vaccine type, e.g. mRNA vaccines) or for multiple vaccines with data stratified by vaccine,

(ii) for an identifiable variant that could be identified as either occurring entirely before the delta wave, or during one of the delta or omicron wave, or with data stratified by variant wave,

(iii) with an identified time since vaccination, and

(iv) be included explicitly in the publication, or be in or readily extractable from the original publication.

Studies (or data within a study) were excluded if they:

(i) Did not present a primary report of vaccine efficacy or effectiveness (e.g. secondary analysis of other studies were excluded as well as review articles, perspectives, opinions)

(ii) Did not report vaccine efficacy or effectiveness compared with an unvaccinated control population (e.g. studies that compared vaccine efficacy/effectiveness of one vaccine against another vaccine)

(iii) Did not report vaccine efficacy or effectiveness over multiple time points since vaccination

(iv) Reported vaccine efficacy/effectiveness for a mixture of vaccines of different types/platforms

(v) Reported vaccine efficacy/effectiveness against a mixture of, or with unspecified, circulating SARS-CoV-2 variants (with the exception of variants occurring before the delta wave, which were allowed to vary).

(vi) Reported vaccine efficacy/effectiveness only for individuals deemed at a high risk of COVID-19 (including studies of exclusively individuals >80 years of age)

We identified 488 potential non-duplicate studies, of which 376 were excluded after screening titles. A total of 112 records were retrieved and assessed. Of these, 96 were subsequently excluded (reasons identified in Figure S9), with a total of 15[1, 2, 3, 4, 5, 6, 7, 8, 9, 10, 11, 12, 13, 14, 15] eligible studies identified and included in our meta-analysis. The complete flow diagram showing the review process is outlined in Figure S9.

Extraction of vaccine effectiveness data

For each identified study we recorded any reported measure of vaccine effectiveness against symptomatic or severe COVID-19 disease. We also recorded the time post vaccination at which these estimates were derived, the vaccine used, the variant against which effectiveness was measured, the age of participants, the type of study and the country in which the study was conducted.

Data was extracted from the eligible studies independently by two of the study co-authors (DC and MS). Data was extracted from identified papers either directly from tables included within the publication, or else was extracted from figures using WebPlotDigitizer[16]. The location within the original publication of the data used for each study is indicated in Table 1. We did not include effectiveness data taken from cohorts classified as at high risk for COVID-19 disease, or from cohorts exclusively comprised of individuals over 80 years of age, as it was felt that these cohorts may have different neutralising antibody responses, and there were not enough cohorts of such a nature to determine this definitively. We classified variants into pre-Delta (predominantly ancestral and Alpha), Delta and Omicron variants. We did not include data reporting on effectiveness exclusively against the Beta variant in the pre-Delta group, as neutralising antibody titres against the Beta variant have previously been identified to be vastly different to those against other pre-Delta variants, and we identified only one study reporting on effectiveness against the Beta variant, meaning that a separate analysis was not possible.

The 15 identified studies that met the above criteria, collectively provided 363 individual data points on vaccine effectiveness against symptomatic or severe COVID-19. The studies included effectiveness for three of the main vaccines used in primary vaccination regimes –

mRNA-1273, BNT162b2 and ChAdOx1-nCoV-19, and on efficacy against symptomatic and severe COVID-19 disease outcomes. These studies are detailed in Table 1.

Multiple Regression Fitting

As described in the main manuscript, we fit a multiple linear regression model to vaccine effectiveness with vaccine, variant and time since vaccination as independent variables.

Estimating Neutralising Antibody Titres

To estimate the mean neutralising antibody titre that would be associated with a given real-world effectiveness estimate, we account for a number of influencing factors. These are:

1) The vaccine that was administered
2) The variant against which effectiveness is being measured
3) The time since vaccination
4) The dosing schedule for the vaccine
5) The timeframe over which efficacy was reported in the original phase 3 trials compared to the time frame measured in the extracted real-world data points.

These factors were included in the estimate as described below.

1) Accounting for the vaccine administered

We have previously estimated the $\log_{10}$ of the starting neutralisation titres observed for a number of vaccines (relative to the geometric mean convalescent titres) using data from phase 1/2 trials for 7 different vaccines[17]. We use these estimates to estimate the peak mean neutralising antibody titres that would have been observed for each vaccine against the wild type variant, using the same dosing schedule as in the relevant phase I/II trials. These estimates are denoted by μ_i (for vaccine i) and are given in Table S2.

2) Accounting for the variant

We have previously estimated the drop in neutralisation titres observed for a number of VoC by combining data from 17 different studies across 5 different vaccines and 5 different variants. This work showed that the fold drop in neutralisation titre (for a given variant) is independent of the vaccine administered[18]. In addition, we have also studied the drop in

neutralisation titre to the Omicron variant[19]. Here we use the fold drop estimated from our previous meta-analysis for the Delta variant (3.9-fold)[18], and the fold drop estimated by Cele et. al. for the Omicron variant (22-fold)[19]. We assume there is no change to the neutralisation titres compared to the ancestral variant for the pre-Delta variants. The fold drops used are denoted by f_j (for variant j) and are specified in Table S2.

3) Accounting for the time since vaccination

To account for waning neutralising antibody levels over the period since vaccination, we assumed that neutralising antibody levels decay exponentially over the trial period according to the formula

$$N_{ab}(t) = N_{ab}(0)e^{-\delta t}. \qquad \text{Equation S1}$$

Where δ corresponds to a half-life of 108 days (estimated in[17], using data from[20]). The distribution for this rate is specified in Table S2.

4) Adjusting for the vaccine dosing schedule

The phase 2 neutralising antibody data used to parameterise the original correlates model for ChAdOx-nCoV-1 was based on a 3 week interval between the first and second vaccine dose. This dosing schedule was subsequently increased to 6-12 weeks in the majority of real world scenarios, resulting in a 50%-90% increase in neutralising antibody levels[21]. In order to account for this discrepancy between the real world implementation and the clinical trial antibody levels we adjusted the neutralising antibody levels for ChAdOx1-nCov vaccinees upwards by a factor of 1.59 fold, to match the estimated increase seen with a 9 week dosing schedule[21]. Therefore we adjusted the antibody levels further by a factor of ϕ_i where

$$\phi_i = \begin{cases} 1.59 & \text{for ChAdOx-nCoV-1} \\ 0 & otherwise \end{cases} \qquad \text{Equation S2}$$

5) Adjusting for duration of follow-up and duration of the original phase 3 trials.

The correlates model[17] was originally fitted to the peak antibody titres seen in the Phase I/II vaccine trials (approximately 2-3 weeks after administration of the final vaccine dose) and the reported vaccine efficacy over the duration of the phase 3 trial randomised control trial (median length of follow up in most phase of the 3 trials was 2 months in line with FDA

requirements[22]). Thus, it essentially reports the relationship between peak neutralising antibody titres at the start of follow up and vaccine protection over the subsequent (approximately) two months of follow up. However, the vaccine effectiveness studies analysed here reported protection over differing periods ranging from 2 weeks to 4 months (i.e. not 2 months in most cases). Differences in the duration of follow-up may confound results due to wanning protection over the course of longer studies. This effect was accounted for by estimating the mean neutralisation level and associated predicted efficacy at the mid-point of follow-up time periods, and using this mid-point in Figures 2, S3 and S4. This mid-point estimation uses the expected antibody decay kinetics from equation S1, to adjust the prediction of efficacy from Khoury et. al.[17], which are based on neutralisation titres at the start of the follow up period. This is essentially an adjustment by a factor of $e^{\delta T}/2$ (where δ is the neutralising antibody decay rate, and T is the trial length). Figures 3A and 3B similarly plot the reported vaccine efficacy at the mid-point of the reported time interval against the estimated neutralising antibody titre (adjusted as above) that should be used to predict efficacy at the mid-point of the interval.

Where a time interval is reported as “more than X weeks / months” we calculate the instantaneous adjusted neutralising antibody titres and associated vaccine effectiveness one month after the lower time bound on this group. i.e. if a study reported efficacy at “more than 5 months” we would correlate this with estimated values at 6 months – one month more than the lower bound of 5 months.

Combined estimate of neutralising antibody levels

The overall neutralising antibody titre is then calculated by incorporating each of the factors described above. Therefore, the neutralising antibody levels calculated for vaccine i against variant j at time t after vaccination, $N_{ab}(i,j,t)$ is given by:

$$N_{ab}(i,j,t) = 10^{\left(\mu_i - f_j + \phi_i + \log_{10} e^{-\delta\left(t-\frac{T}{2}\right)}\right)}. \qquad \text{Equation S3}$$

Where μ_i represents the mean of the $\log_{10}$ of the neutralising antibody levels against ancestral virus for subjects vaccinated with vaccine i, f_j represents the fold drop in

neutralising antibody titre for variant j (compared to ancestral virus), δ is the decay rate of neutralising antibodies, and T is the average length of the original phase III trials.

As all of the factors determining the neutralisation titre for a given vaccine regimen at a given time each contain their own confidence bands, the cumulative effect of these confidence bands is used to calculate the overall uncertainty in the neutralising antibody levels (see next section and Table S2).

Predicting vaccine effectiveness using the previously published correlates model

We have previously developed and fitted a model correlating neutralising antibody titres (immunogenicity data taken from phase I/II trials) to vaccine efficacy (protective efficacy taken from phase III trials) against symptomatic and severe SARS-CoV-2 infection[17]. This published model was parameterised using data from seven published studies of vaccine efficacy along with data on protection from COVID-19 after previous infection. Specifically, vaccine effectiveness, VE, is defined as:

$$VE(\mu_i, f_j) = \int_{-\infty}^{\infty} N(x, \mu_i - f_j\,, \sigma)\,\frac{1}{1+e^{-k(x-x_{50})}}\,dx \qquad \text{Equation S4}$$

Where $\sigma = 0.46$, $k = 3.1$ and $x_{50} = \log_{10} 0.2$ for symptomatic infection and $x_{50} = \log_{10} 0.03$ for severe infection[17, 23] (Table S3). Where, as above, μ_i, represents the mean of the $\log_{10}$ of the neutralising antibody titres for vaccine i against the ancestral strain of the virus, and f_j represents the fold decrease in neutralising antibody titres for variant *j*. Values of these parameters were collected from the literature as outlined in Tables S3 and S4. In this work, references to the “correlates model” refer to the use of this model, as originally published and parameterised. I.e. the parameters of this model were not re-estimated or fitted in this study, but were used as originally reported[17].

Determining confidence intervals using parametric bootstrapping

Confidence intervals of all estimates for neutralising antibody titres and predicted efficacies (shaded regions) in Figures 2, 3, S1-S4 were generated using parametric bootstrapping on the parameters with uncertainty in their estimation (as previously reported in reference[18], parameters given in Tables S3 and S4) as follows. For any time point along the x-axis in Figures 2, S3 and S4, or for the horizontal placement of data points in Figures 3A and B, the mean neutralising antibody titre was first estimated using equation S3. Then the confidence

bands of the neutralising antibody titres was estimated by repeatedly using equation S3 to re-estimate the neutralising antibody titre, while sampling parameters from the distributions given in Table S2.

Subsequently, for any neutralisation ratio either calculated above, or for a position on the x-axis in Figure 3A or B, equation S4 was first used to estimate the mean corresponding protective efficacy. Then the distribution of efficacies was estimated by repeating the efficacy calculation with equation S4, using parameter values drawn randomly from distributions according to their standard error or covariance matrix (normal and bivariant normal distributions respectively in Table S3).

Sampling was performed 100,000 times and the lower confidence bound was estimated from at the 2.5% percentile, while the upper confidence bound was taken from the 97.5% percentile.

Supplementary Tables

Parameter	Description	Vaccine / Variant	Effectiveness reduction value (95% CI)*
A_i	Vaccine Specific Efficacy difference	mRNA-1273	reference
		BNT162b2	1.6 (-0.1 - 3.3)
		Any mRNA	1.5 (-1.0 – 4.0)
		ChAdOx1 nCov-19	6.2 (3.8 - 8.6)
B_j	Variant Specific Efficacy difference	pre-Delta	reference
		Delta	-3.9 (-6.1 - -1.6)
		Omicron	31.4 (27 - 35.8)
C_j	Loss in efficacy per month since vaccination	all	1.7 (1.4 - 2.1)
ζ_{study}	Random effect for study	Distribution	$N(0, 5.8^2)$
Eff_{base}	Reference Efficacy	Baseline	96.6 (92.1 - 101)

Table S1 Parameters estimated in the multiple regression model fitting for severe COVID-19. *Note that a positive value indicates a lower estimated efficacy as the coefficients of equation 1 have a negative sign in front of them. $N(0, \sigma^2)$ represents a normal distribution with mean of 0 and a standard deviation of σ.

Parameter	Description	Vaccine / Variant	Mean Value	Distribution	Reference
μ_i	Starting neutralising antibody levels (as a fold of convalescent)	mRNA-1273	$\log_{10} 4.1$	$N(\log_{10} 4.1\,, .006)$	[17]
		BNT162b2	$\log_{10} 2.4$	$N\ (\log_{10} 2.4\,, .01)$	[17]
		ChAdOx1 nCov-19	$\log_{10} 0.8$	$N(\log_{10} 0.8\,, .018)$	[17]
		Any mRNA	$\log_{10} 3.1$	$N(\log_{10} 3.1\,, .023)$	(geometric mean of mRNA-1273 and BNT162b2)
f_j	Fold change in neutralisation titre against variant	Delta	$-\log_{10} 3.9$	$N(-\log_{10} 3.9\,, .003)$	[18]
		Omicron	$-\log_{10} 22$	$N(-\log_{10} 22\,, .005)$	[19]
δ	Neutralising antibody decay rate	N/A	$\ln 2/108$	$N(6.42, .001) * 10^{-3}$	[17, 20]
T	Phase 3 clinical trial length	N/A	60 days	N/A	[24, 25, 26]
ϕ_i	Dosage adjustment for different ChAdOx1-nCoV-19 dose interval	ChAdOx-nCoV-1	$\log_{10} 1.59$	N/A	[21]
		Other vaccines	0	N/A	N/A

Table S2 Parameters used in estimating neutralisation titre. The GMT value used for mRNA is the geometric mean value of mRNA-1273 and BNT162b2 values. Note that all these parameters were estimated from previously published work.

Parameter	Description	Endpoint	Mean Value	Distribution	Reference
σ		N/A	0.46	$N(0.465,0.022)$	[17]
k	Hill coefficient*	Symptomatic	$e^{1.13}$	$e^{N(1.13,.031)}$	[17]
		Severe	$e^{1.12}$	$e^{N(1.12,.03)}$	[17]
x_{50}	IC50 for protection against disease*	Symptomatic	$\log_{10} 0.20$	$N(\log_{10} 0.20\,, 0.006)$	[17]
		Severe	$\log_{10} 0.03$	$N(\log_{10} 0.03\,, 0.099)$	[17]

Table S3 Model parameters used in estimating the relationship between neutralising antibody titre and protection from COVID-19 (taken from Khoury et. al. [17]) *Note that the hill coefficient and IC50 parameters are selected from a bivariate normal distribution with covariance matrix given by $C = \begin{pmatrix} .031 & .011 \\ .011 & .006 \end{pmatrix}$ for symptomatic protection and $C = \begin{pmatrix} .03 & .03 \\ .03 & .099 \end{pmatrix}$ for severe protection.

References

1. Bianchi FP*, et al.* BNT162b2 mRNA COVID-19 Vaccine Effectiveness in the Prevention of SARS-CoV-2 Infection and Symptomatic Disease in Five-Month Follow-Up: A Retrospective Cohort Study. *Vaccines (Basel)* **9**, (2021).

2. Katikireddi SV*, et al.* Two-dose ChAdOx1 nCoV-19 vaccine protection against COVID-19 hospital admissions and deaths over time: a retrospective, population-based cohort study in Scotland and Brazil. *Lancet* **399**, 25-35 (2022).

3. Thomas SJ*, et al.* Safety and Efficacy of the BNT162b2 mRNA Covid-19 Vaccine through 6 Months. *N Engl J Med* **385**, 1761-1773 (2021).

4. Skowronski DM*, et al.* Two-dose SARS-CoV-2 vaccine effectiveness with mixed schedules and extended dosing intervals: test-negative design studies from British Columbia and Quebec, Canada. *medrxiv*, (2021).

5. Ferdinands JM*, et al.* Waning 2-Dose and 3-Dose Effectiveness of mRNA Vaccines Against COVID-19-Associated Emergency Department and Urgent Care Encounters and Hospitalizations Among Adults During Periods of Delta and Omicron Variant Predominance - VISION Network, 10 States, August 2021-January 2022. *MMWR Morb Mortal Wkly Rep* **71**, 255-263 (2022).

6. Thompson MG*, et al.* Effectiveness of a Third Dose of mRNA Vaccines Against COVID-19-Associated Emergency Department and Urgent Care Encounters and Hospitalizations Among Adults During Periods of Delta and Omicron Variant Predominance - VISION Network, 10 States, August 2021-January 2022. *MMWR Morb Mortal Wkly Rep* **71**, 139-145 (2022).

7. Andrews N*, et al.* Covid-19 Vaccine Effectiveness against the Omicron (B.1.1.529) Variant. *N Engl J Med* **386**, 1532-1546 (2022).

8. Andrews N*, et al.* Duration of Protection against Mild and Severe Disease by Covid-19 Vaccines. *N Engl J Med* **386**, 340-350 (2022).

9. Chemaitelly H*, et al.* Waning of BNT162b2 Vaccine Protection against SARS-CoV-2 Infection in Qatar. *N Engl J Med* **385**, e83 (2021).

10. Tseng HF*, et al.* Effectiveness of mRNA-1273 against SARS-CoV-2 Omicron and Delta variants. *Nat Med*, (2022).

11. El Sahly HM*, et al.* Efficacy of the mRNA-1273 SARS-CoV-2 Vaccine at Completion of Blinded Phase. *N Engl J Med* **385**, 1774-1785 (2021).

12. Goldberg Y*, et al.* Waning Immunity after the BNT162b2 Vaccine in Israel. *N Engl J Med* **385**, e85 (2021).

13. Rosenberg ES*, et al.* Covid-19 Vaccine Effectiveness in New York State. *N Engl J Med* **386**, 116-127 (2022).

14. Keehner J*, et al.* Resurgence of SARS-CoV-2 Infection in a Highly Vaccinated Health System Workforce. *N Engl J Med* **385**, 1330-1332 (2021).

15. Poukka E*, et al.* Cohort study of Covid-19 vaccine effectiveness among healthcare workers in Finland, December 2020 - October 2021. *Vaccine* **40**, 701-705 (2022).

16. Rohatgi A. WebPlotDigitizer Ver 4.5. https://automeris.io/WebPlotDigitizer (2022).

17. Khoury DS*, et al.* Neutralizing antibody levels are highly predictive of immune protection from symptomatic SARS-CoV-2 infection. *Nat Med* **27**, 1205-1211 (2021).

18. Cromer D*, et al.* Neutralising antibody titres as predictors of protection against SARS-CoV-2 variants and the impact of boosting: a meta-analysis. *Lancet Microbe* **3**, e52-e61 (2022).

19. Cele S*, et al.* Omicron extensively but incompletely escapes Pfizer BNT162b2 neutralization. *Nature* **602**, 654-656 (2022).

20. Dan JM*, et al.* Immunological memory to SARS-CoV-2 assessed for up to 8 months after infection. *Science* **371**, (2021).

21. Voysey M*, et al.* Single-dose administration and the influence of the timing of the booster dose on immunogenicity and efficacy of ChAdOx1 nCoV-19 (AZD1222) vaccine: a pooled analysis of four randomised trials. *Lancet* **397**, 881-891 (2021).

22. FDA. Emergency Use Authorization for Vaccines to Prevent COVID-19.).

23. Khoury DS*, et al.* R Code: Neutralizing antibody levels are highly predictive of immune protection from symptomatic SARS-CoV-2 infection Ver 2021/05/19. https://github.com/InfectionAnalytics/COVID19-ProtectiveThreshold (2021).

24. Voysey M*, et al.* Safety and efficacy of the ChAdOx1 nCoV-19 vaccine (AZD1222) against SARS-CoV-2: an interim analysis of four randomised controlled trials in Brazil, South Africa, and the UK. *Lancet* **397**, 99-111 (2021).

25. Polack FP*, et al.* Safety and Efficacy of the BNT162b2 mRNA Covid-19 Vaccine. *N Engl J Med* **383**, 2603-2615 (2020).

26. Baden LR*, et al.* Efficacy and Safety of the mRNA-1273 SARS-CoV-2 Vaccine. *N Engl J Med* **384**, 403-416 (2021).

Supplementary Figures

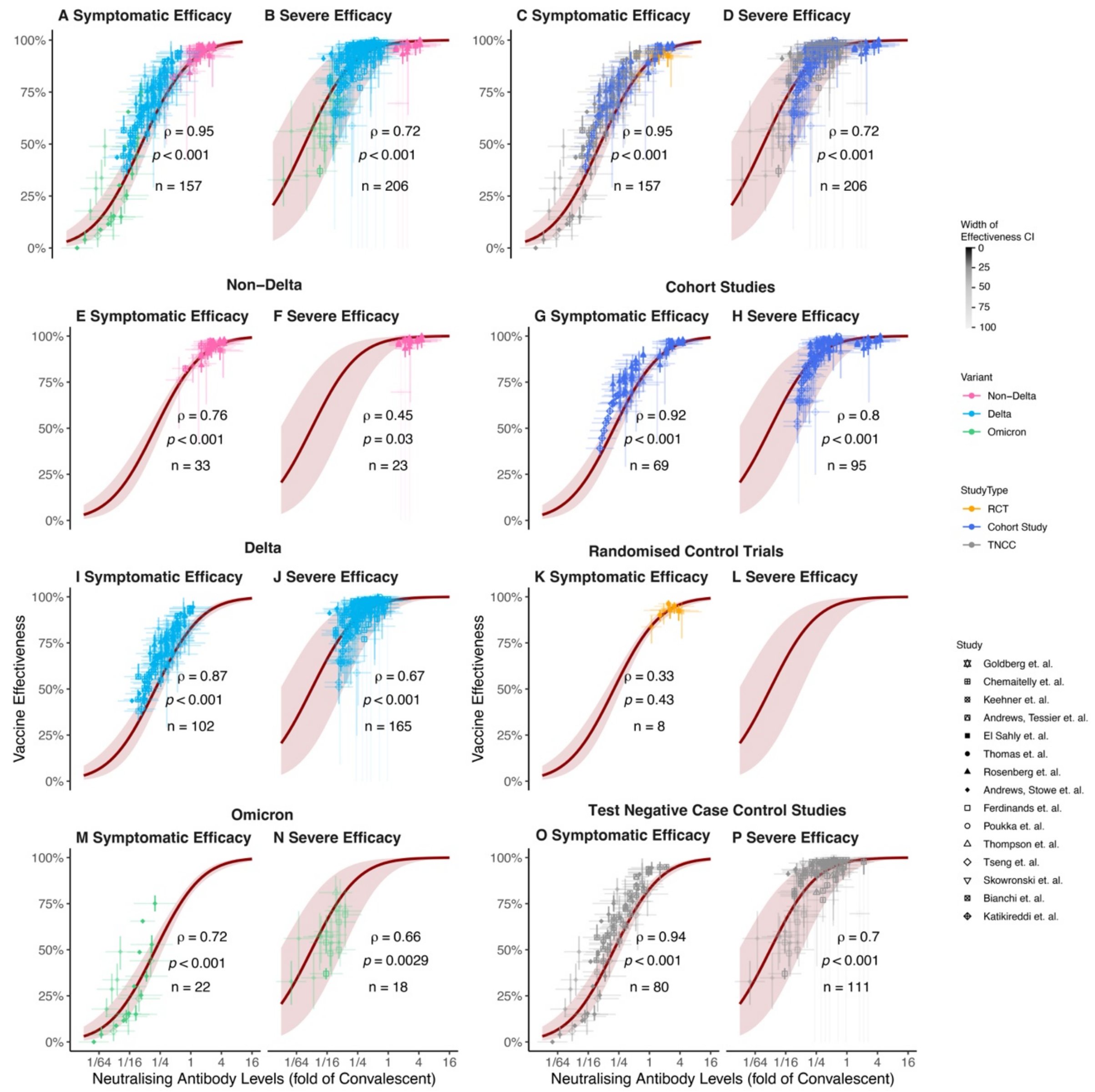

Supplementary Figure 1 Correlation between estimated neutralising antibody titres and vaccine effectiveness stratified by variant and clinical trial type

Correlation between estimated neutralising antibody titres (accounting for vaccine used, variant studied and time since vaccination) and clinical data for vaccine effectiveness against symptomatic and severe COVID-19, coloured by variant (left hand side, panels A,B,E,F,I,J,M,N, effectiveness for pre-Delta variants shown in pink, the Delta variant shown in blue and the Omicron variant shown in green) and study type (right hand side, panels C,D,G,H,K,L,O,P, effectiveness for randomised control trials (RCT) variants shown in orange,

cohort studies shown in purple and test negative case control studies (TNCC) shown in green). Top row includes all data, bottom three rows include data split by variant (left two columns) and study type (right two columns). Numbers show ρ and p-value for Spearman correlations and the number of data-points shown in each plot. Note that our meta-analysis did not include any additional data for severe disease in a randomised control trial and so there is no extracted data shown in panel (L). Panels A shows effectiveness against variants.

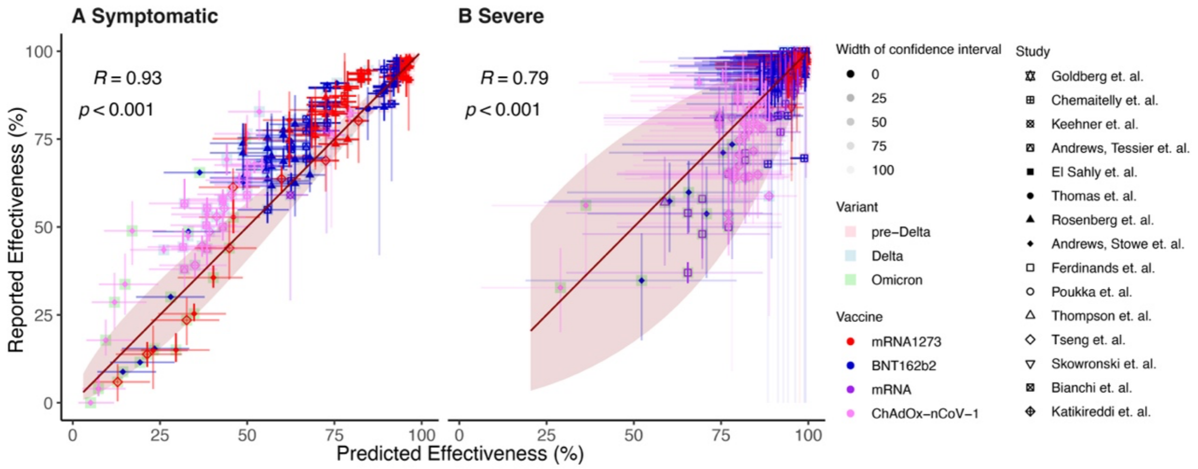

Supplementary Figure 2 Correlation between predicted effectiveness and reported effectiveness

Comparison between predicted efficacy for a specific vaccine, variant and timepoint (x-axis) and the corresponding observed effectiveness estimate from the meta-analysis (y-axis) for (A) symptomatic and (B) severe COVID-19. Dark red line shows 1:1 relationship, and red band shows the 95% confidence intervals (as determined by parametric bootstrapping). Figure shows effectiveness following mRNA1273 (red), BNT162b2 (blue) any mRNA (purple) and ChAdOx-nCov-1 (pink) vaccination against pre-Delta (pink background) Delta (blue background) and Omicron (green background) variants. Numbers show R-value and p-value for Pearson correlations.

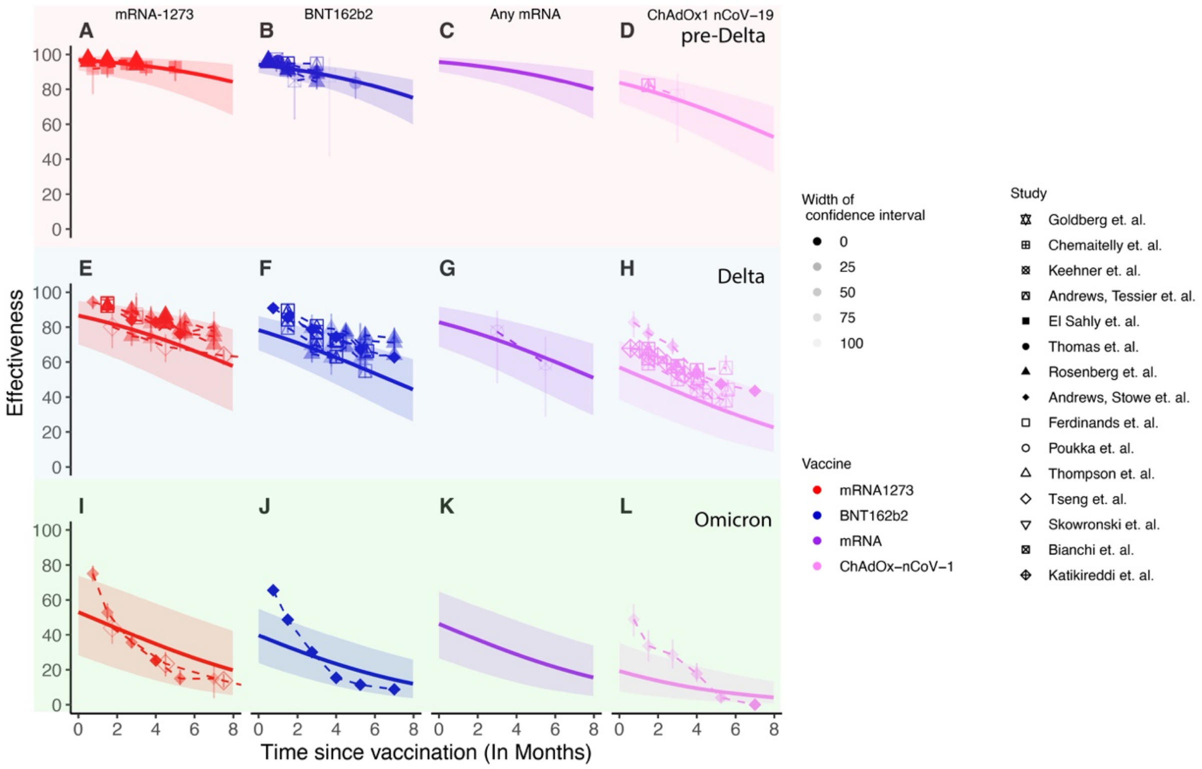

Supplementary Figure 3 Model estimates for vaccine effectiveness against symptomatic SARS-CoV-2 infection shown along with data extracted from our systematic review

Model estimates (solid lines) and 95% confidence intervals (shaded area, as determined by parametric bootstrapping) for vaccine effectiveness against symptomatic SARS-CoV-2 infection with pre-Delta (top row, panels A-D), Delta (middle row, panels E-H) and Omicron (bottom row, panels I-L) variants. Data extracted from the clinical studies of vaccine effectiveness are overlaid as points (whiskers indicate 95% CI). Note that for panels C and K no effectiveness data was available. Model parameters and distributions are taken from references [17, 18, 19] and outlined in the supplementary methods and Tables S2 and S3. Figure shows effectiveness following mRNA1273 (red), BNT162b2 (blue) any mRNA (purple) and ChAdOx-nCov-1 (pink) vaccination.

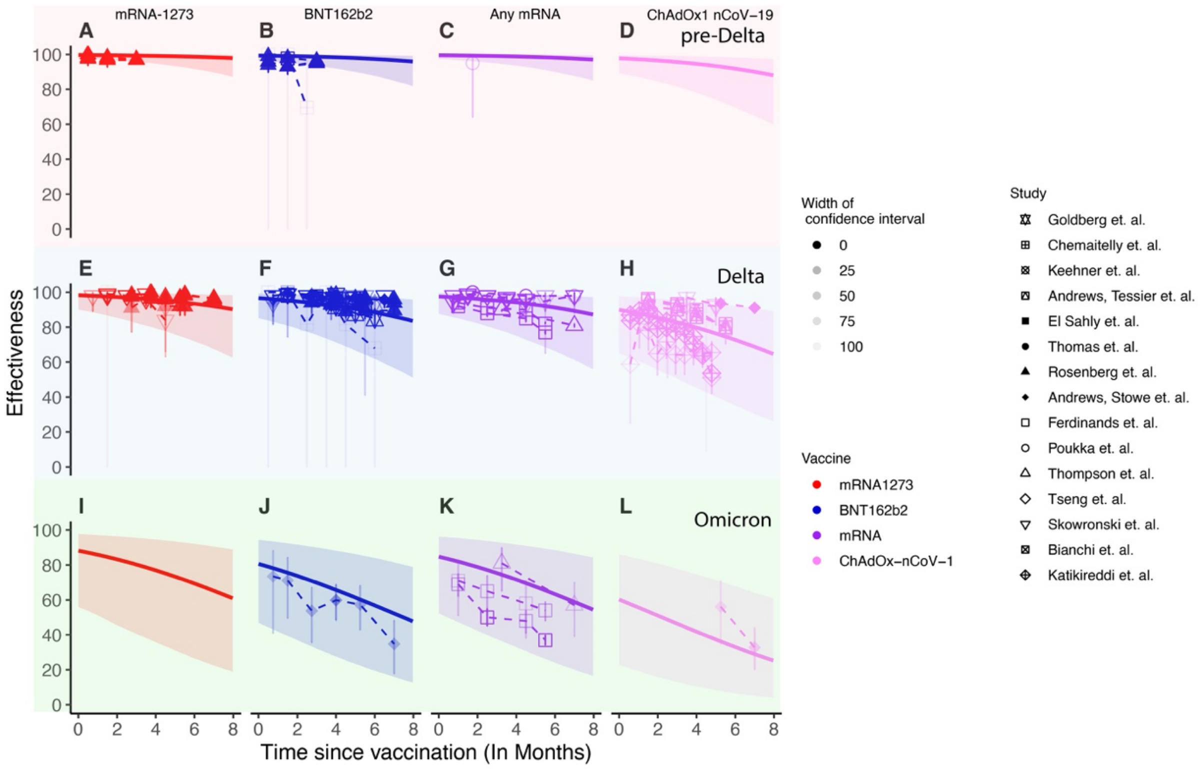

Supplementary Figure 4 Model estimates for vaccine effectiveness against severe SARS-CoV-2 infection shown along with data extracted from our systematic review

Model estimates (solid lines) and 95% confidence intervals (shaded area, as determined by parametric bootstrapping) for vaccine effectiveness against severe SARS-CoV-2 infection with pre-Delta (top row, panels A-D), Delta (middle row, panels E-H) and Omicron (bottom row, panels I-L) variants. Data extracted from the clinical studies of vaccine effectiveness are overlaid as points (whiskers indicate 95% CI). Note that for panels D and I no effectiveness data was available. Model parameters and distributions are taken from references [17, 18, 19] and outlined in the supplementary methods and Tables S2 and S3. Figure shows effectiveness following mRNA1273 (red), BNT162b2 (blue) any mRNA (purple) and ChAdOx-nCov-1 (pink) vaccination.

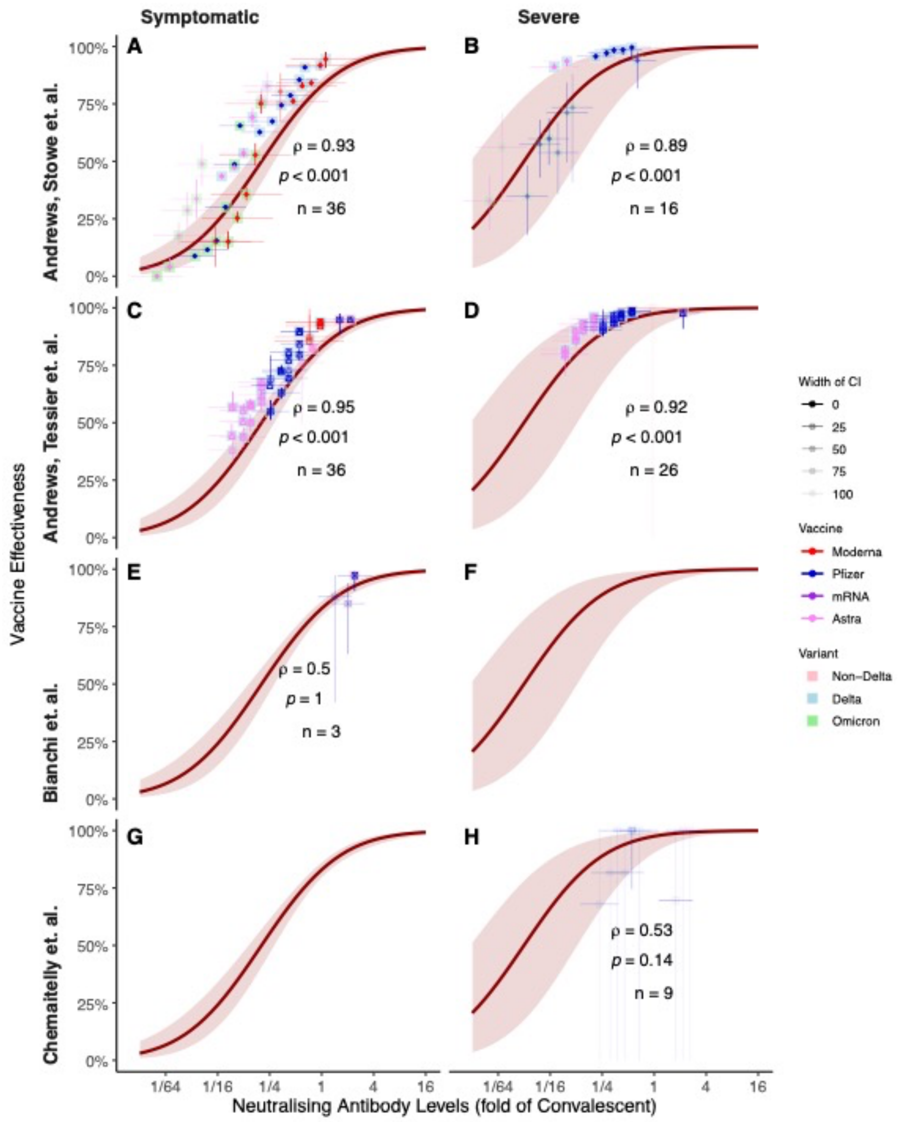

Supplementary Figure 5 Correlation between estimated neutralising antibody titres and vaccine effectiveness stratified by study

Correlation between estimated neutralising antibody titres (accounting for vaccine used, variant studied and time since vaccination) and clinical data for vaccine effectiveness against symptomatic and severe COVID-19, coloured by variant and split by study for data from studies[1, 7, 8, 9]. Numbers show ρ and p-value for Spearman correlations and the number of data-points shown in each plot. Note that some studies did not contain effectiveness data for both symptomatic and severe outcomes, and hence some of the plot panels are blank. Figure shows effectiveness following mRNA1273 (red), BNT162b2 (blue) any mRNA (purple) and ChAdOx-nCov-1 (pink) vaccination against pre-Delta (pink background) Delta (blue background) and Omicron (green background) variants.

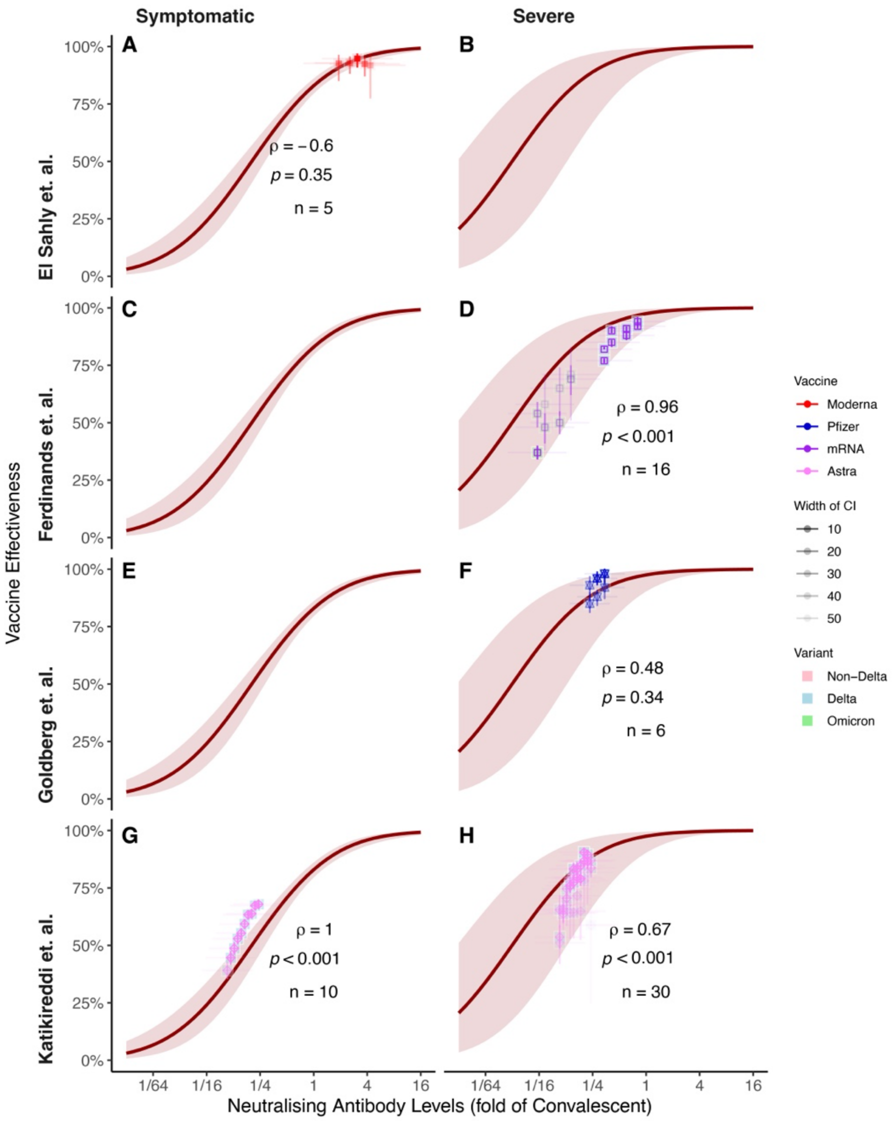

Supplementary Figure 6 Correlation between estimated neutralising antibody titres and vaccine effectiveness stratified by study

Correlation between estimated neutralising antibody titres (accounting for vaccine used, variant studied and time since vaccination) and clinical data for vaccine effectiveness against symptomatic and severe COVID-19, coloured by variant and split by study for data from studies[2, 5, 11, 12]. Numbers show ρ and p-value for Spearman correlations and the number of data-points shown in each plot. Note that some studies did not contain effectiveness data for both symptomatic and severe outcomes, and hence some of the plot panels are blank. Figure shows effectiveness following mRNA1273 (red), BNT162b2 (blue) any mRNA (purple) and ChAdOx-nCov-1 (pink) vaccination against pre-Delta (pink background) Delta (blue background) and Omicron (green background) variants.

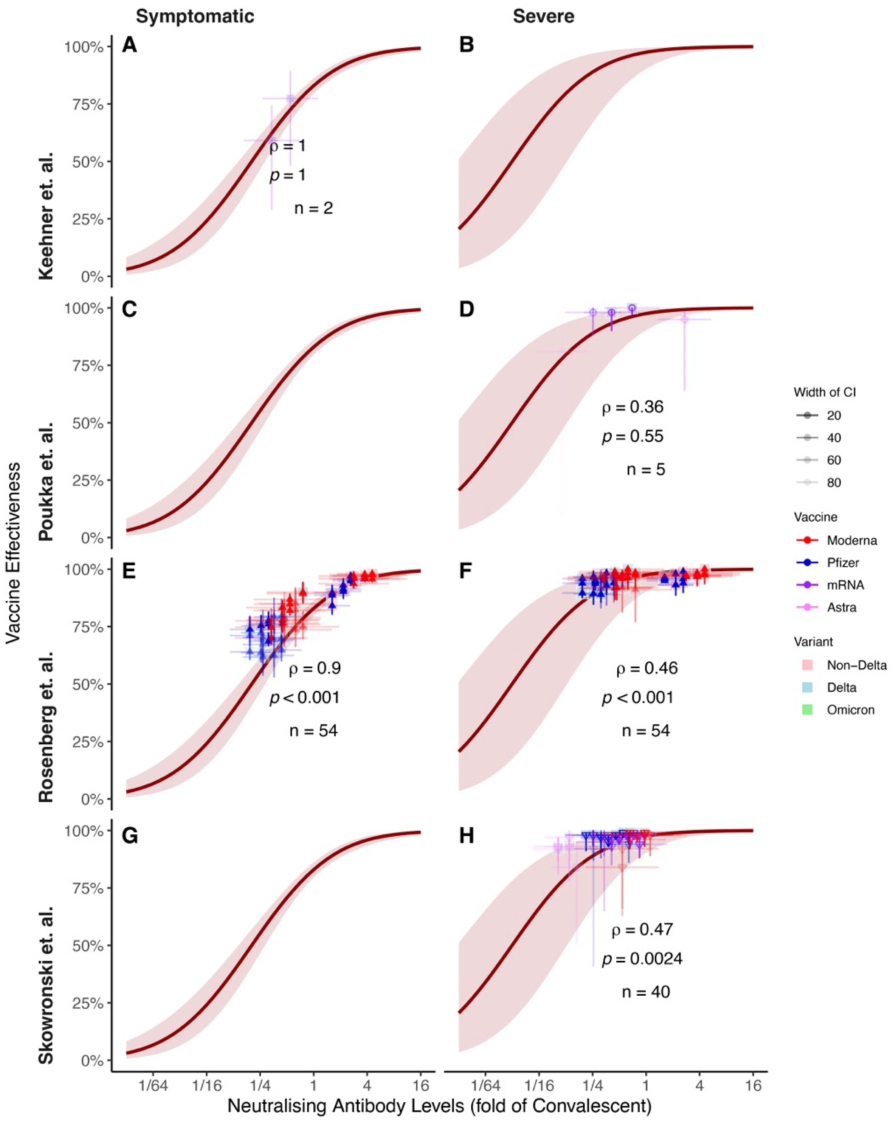

Supplementary Figure 7 Correlation between estimated neutralising antibody titres and vaccine effectiveness stratified by study

Correlation between estimated neutralising antibody titres (accounting for vaccine used, variant studied and time since vaccination) and clinical data for vaccine effectiveness against symptomatic and severe COVID-19, coloured by variant and split by study for data from studies[4, 13, 14, 15]. Numbers show ρ and p-value for Spearman correlations and the number of data-points shown in each plot. Note that some studies did not contain effectiveness data for both symptomatic and severe outcomes, and hence some of the plot panels are blank. Figure shows effectiveness following mRNA1273 (red), BNT162b2 (blue) any mRNA (purple) and ChAdOx-nCov-1 (pink) vaccination against pre-Delta (pink background) Delta (blue background) and Omicron (green background) variants.

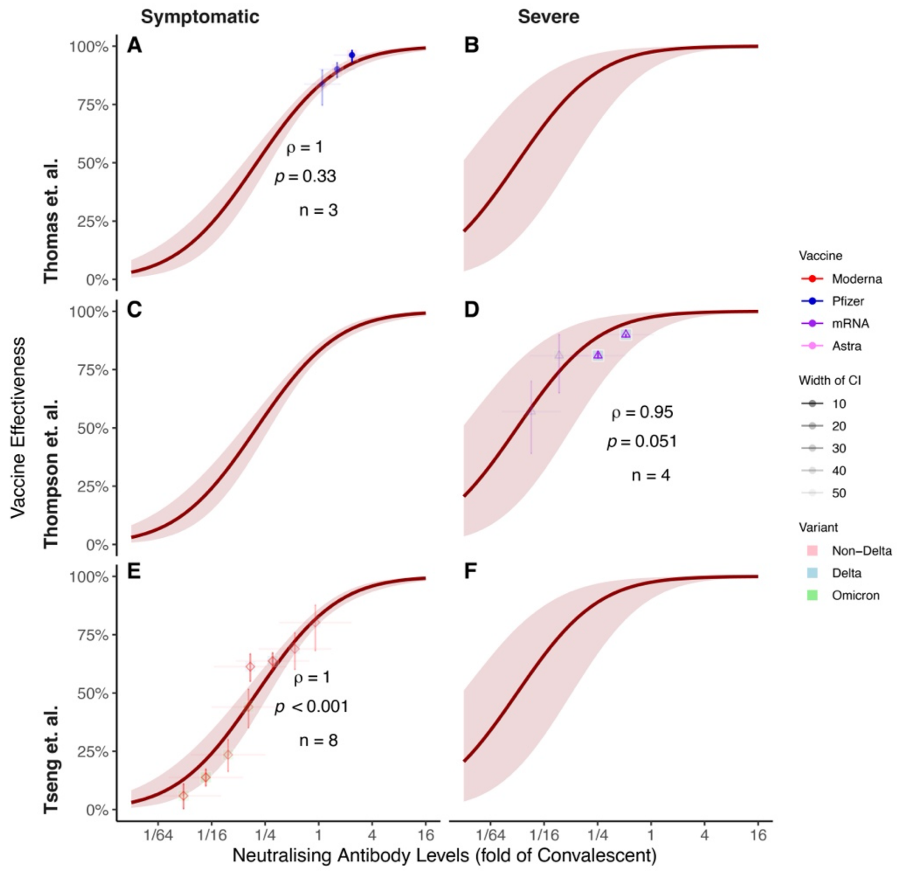

Supplementary Figure 8 Correlation between estimated neutralising antibody titres and vaccine effectiveness stratified by study

Correlation between estimated neutralising antibody titres (accounting for vaccine used, variant studied and time since vaccination) and clinical data for vaccine effectiveness against symptomatic and severe COVID-19, coloured by variant and split by study for data from studies[3, 6, 10]. Numbers show ρ and p-value for Spearman correlations and the number of data-points shown in each plot. Note that some studies did not contain effectiveness data for both symptomatic and severe outcomes, and hence some of the plot panels are blank. Figure shows effectiveness following mRNA1273 (red), BNT162b2 (blue) any mRNA (purple) and ChAdOx-nCov-1 (pink) vaccination against pre-Delta (pink background) Delta (blue background) and Omicron (green background) variants.

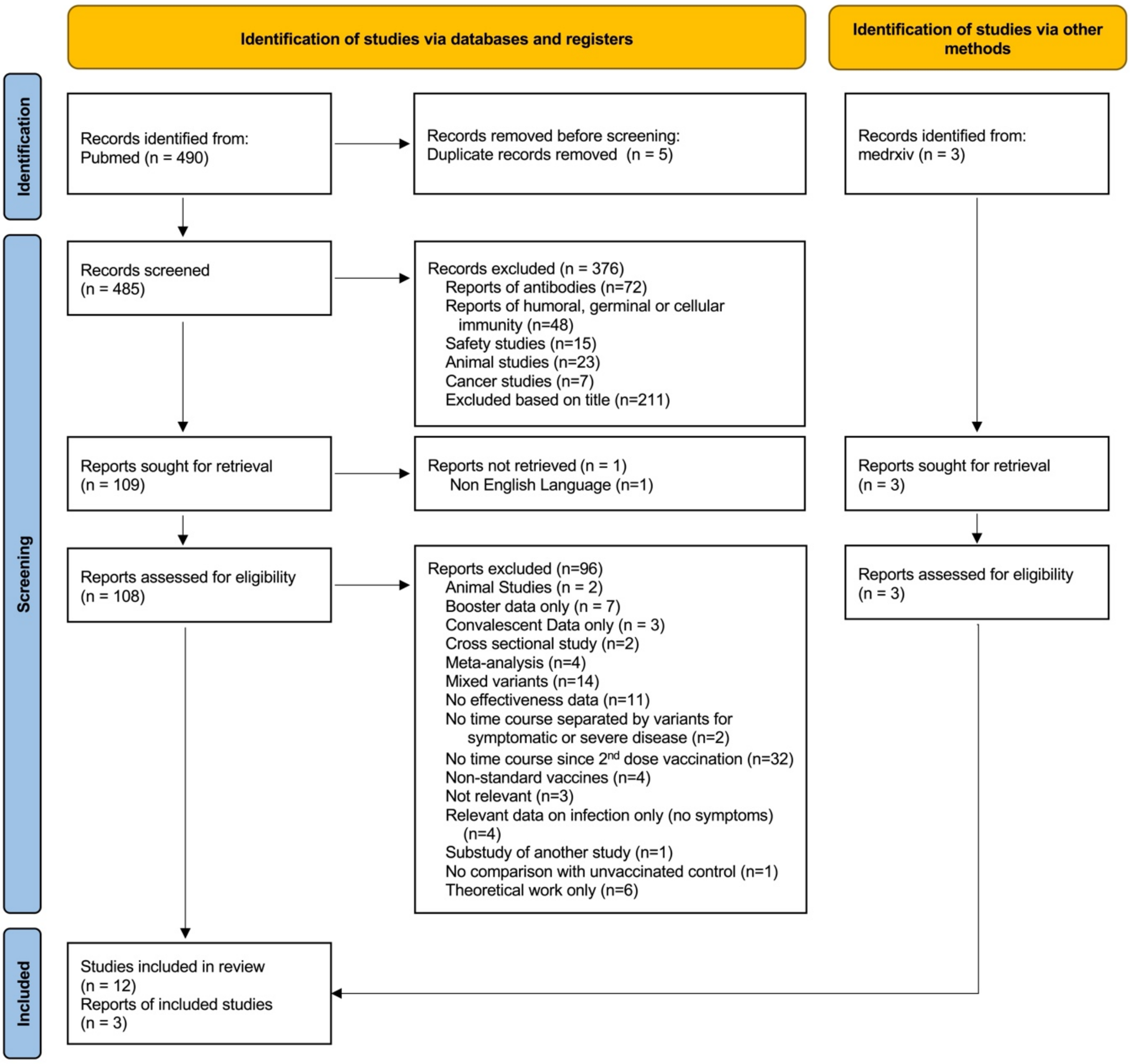

445 **Supplementary Figure 9 PRISMA Flow diagram showing selection process of studies included in the analysis.**
